# Evaluation of Potential Peptide-Based Inhibitors against SARS-CoV-2 and Variants of Concern

**DOI:** 10.1155/2023/3892370

**Published:** 2023-10-13

**Authors:** Hattan Boshah, Faris Samkari, Alexander U. Valle-Pérez, Sarah M. Alsawaf, Ali H. Aldoukhi, Panayiotis Bilalis, Salwa A. Alshehri, Hepi H. Susapto, Charlotte A. E. Hauser

**Affiliations:** ^1^Laboratory for Nanomedicine, Division of Biological and Environmental Science and Engineering (BESE), King Abdullah University of Science and Technology (KAUST), Thuwal 23955-6900, Saudi Arabia; ^2^KAUST Smart Health Initiative (KSHI), King Abdullah University of Science and Technology (KAUST), Thuwal 23955-6900, Saudi Arabia; ^3^Computational Bioscience Research Center (CBRC), King Abdullah University of Science and Technology (KAUST), Thuwal 23955-6900, Saudi Arabia; ^4^Red Sea Research Center (RSRC), King Abdullah University of Science and Technology (KAUST), Thuwal 23955, Saudi Arabia; ^5^Department of Biochemistry, Faculty of Science, University of Jeddah, Jeddah, Saudi Arabia

## Abstract

The severe acute respiratory syndrome coronavirus 2 (SARS-CoV-2) pandemic has greatly affected all aspect of life. Although several vaccines and pharmaceuticals have been developed against SARS-CoV-2, the emergence of mutated variants has raised several concerns. The angiotensin-converting enzyme (ACE2) receptor cell entry mechanism of this virus has not changed despite the vast mutation in emerging variants. Inhibiting the spike protein by which the virus identifies the host ACE2 receptor is a promising therapeutic countermeasure to keep pace with rapidly emerging variants. Here, we synthesized two ACE2-derived peptides, P1 and P25, to target and potentially inhibit SARS-CoV-2 cell entry. These peptides were evaluated *in vitro* using pseudoviruses that contained the SARS-CoV-2 original spike protein, the Delta-mutated spike protein, or the Omicron spike protein. An in silico investigation was also done for these peptides to evaluate the interaction of the synthesized peptides and the SARS-CoV-2 variants. The P25 peptide showed a promising inhibition potency against the tested pseudoviruses and an even higher inhibition against the Omicron variant. The IC_50_ of the Omicron variant was 60.8 *μ*M, while the IC_50_s of the SARS-CoV-2 original strain and the Delta variant were 455.2 *μ*M and 546.4 *μ*M, respectively. The *in silico* experiments also showed that the amino acid composition design and structure of P25 boosted the interaction with the spike protein. These findings suggest that ACE2-derived peptides, such as P25, have the potential to inhibit SARS-CoV-2 cell entry in vitro. However, further in vivo studies are needed to confirm their therapeutic efficacy against emerging variants.

## 1. Introduction

The severe acute respiratory syndrome coronavirus 2 (SARS-CoV-2) has significantly impacted the human health, global economy, and society. Such an impact will require years for complete recovery. In December 2019, this virus was recognized as the causative agent responsible for the COVID-19 pandemic [1]. As of September 2022, there have been over 605 million confirmed cases and at least 6.4 million global deaths [2]. Due to the major efforts in vaccine development, the virus fatality decreased significantly [3]. However, the virus's rapid mutations and the risk of the mutated virus evading immunity are still immense threats. Due to this potential health hazard, research regarding this pandemic is of the utmost importance.

The earliest variant of concern (VOC), alpha, was reported in December 2020 [4]. This variant, which has potent substitution mutations such as D614G and N501Y, represents a fitness advantage that enhances viral entry into the target cell [5–7]. Additional mutations and deletions were reported later in several VOCs, and these VOCs were noted to have high viral transmissibility, binding affinity, and antibody escape [8]. For example, the Omicron variant, which is one of the most dominant strains of SARS-CoV-2 worldwide, has over 32 mutations clustered within the receptor binding domain (RBD) and the spike protein's N-terminal domains, which are the primary targets of neutralizing antibodies [9, 10]. Therefore, this variant is likely to escape the neutralizing effect of antibodies, thereby reducing the efficiency of the otherwise highly effective vaccines.

While vaccines are the most effective prevention method, antiviral drugs remain necessary to decrease the severity of the infection in critical cases. Several studies have tested the therapeutic potential of SARS-CoV-2 nonspecific treatments, such as HIV protease inhibitors, influenza neuraminidase inhibitors, and other nucleoside reverse transcriptase inhibitors [11–13]. Additionally, several protein therapeutic approaches have been explored as a potential SARS-CoV-2-specific treatment. These included the use of full-length recombinant human ACE2 protein [14] and recombinant SARS-CoV-2 RBD protein [15].

Peptide-based therapeutics is another attractive approach that can be used as a SARS-CoV-2-specific treatment. Peptide inhibitors for the virus can be designed to act as binding inhibitors by targeting the host ACE2 receptor binding site or the receptor binding domain of the virus spike protein. Alternatively, inhibition can also be achieved using fusion inhibitor peptides that interfere with the formation of the fusion core during viral infection [16–18]. The sequence of such peptides can be driven either from the ACE2 or the spike protein itself. Xia et al. tested the inhibitory effect of multiple spike-driven peptides that target the HR1 domain of SARS-CoV-2 [19]. The *in silico* analysis and molecular dynamic studies revealed other ACE2-driven peptides with a high inhibitory potential of SARS-CoV-2 infection. However, the inhibitory activity and binding affinities of such inhibitors were never validated on the original virus or other VOCs.

The molecular docking simulation and analysis of the RBD-ACE2 cocrystal structure by Zhang et al. revealed a potential 23-mer peptide inhibitor driven by the human ACE2 protein [20]. However, *in vitro* testing of the reported sequence repeatedly proved the inability of the peptide to inhibit viral entry to ACE2-expressing cells [14]. Therefore, further docking analysis and virtual screening of the same peptide resulted in a modified sequence with mutated residues to improve the overall activity. The reported simulation of the modified peptide suggested stronger interaction with the spike protein of the original SARS-CoV-2 virus [21].

Despite its potentials, the sequence has not yet been experimentally validated against the original virus and emerging variants.

**Figure 1:**
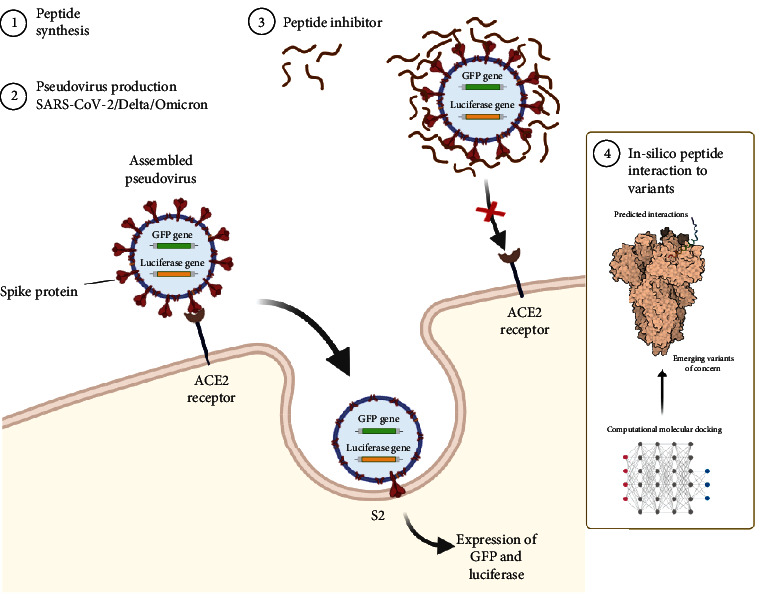
Schematic of pseudovirus entry inhibition using rationally designed peptide inhibitors.

In this study, we investigated and evaluated the binding affinities and inhibitory activity of a previously reported ACE2-derived peptide, P1, and a modified sequence of the same region, P25, which are predicted by Panda et al. and referred to as peptide #13 in their study [20, 21]. Based on their computational study, P25 is claimed to show a stronger binding affinity than P1, which was found to be ineffective in previous experimental studies, and can provide a better inhibition against SARS-CoV-2. However, the efficacy of P25 was only supported by simulations. We aimed to provide experimental data to challenge these claims and elucidate if these same peptides can bind and inhibit the SARS-CoV-2-associated variants of concern, such as Omicron and Delta. Peptide inhibition activities were tested against the pseudovirus of the SARS-CoV-2 virus, as well as the Delta and the Omicron variants, as shown in Figure 1. The computational analysis of these peptides was also performed to predict the behavior of interactions against different VOCs. This study highlights the relevance of peptide-based inhibitors as a potential antiviral alternative to mitigate emerging variants and provide a rapid response against them.

## 2. Materials and Methods

For the peptide synthesis, diethyl ether, acetonitrile, formic acid, N,N-dimethylformamide (DMF), trifluoroacetic acid (TFA), triisopropylsilane (TIS), diisopropylethylamine (DIPEA), dichloromethane (DCM), and piperidine were obtained from Sigma-Aldrich. In addition, 2-(1H-benzotriazole-1-yl)-1,1,3,3-tetramethyluronium hexafluorophosphate (TBTU), hydroxybenzotriazole (HOBt), MBHA rink amide resin, and 9-fluorenylmethoxycarbonyl- (Fmoc-) protected amino acids were acquired from GL Biochem, China. All compounds were used in their original state without purification or modification.

For MST, recombinant SARS-CoV-2 spike S1 protein and human ACE2 protein were purchased from Sino Biological (Cat: 40591-V08H, Cat: 10108-H08B). For ELISA, maleic anhydride-activated plate, REF 15100, ELISA blocking buffer, N502, and ACE-2 antibody were obtained from Thermo Scientific. Carbonate-bicarbonate buffer capsule, C3041-100CAP, and Tween-20, T2700-500ML, were acquired from Sigma-Aldrich. Phosphate-buffered saline was obtained from Fisher Scientific (096292) and S1 protein from Sino Biological (40591-V08H). Both TMB substrate, ab171523, and spike antibody, ab273073, were provided by Abcam. ELISA stop solution was acquired from Invitrogen (SS04) and Thermo Scientific (PA5-20045), anti-rabbit secondary antibody from Abcam (ab6721), and anti-human secondary antibody from Abcam (ab99759).

For cell culture and inhibition assay, ACE2-expressing cells (293T ACE2 SSC22) and all plasmids were generously provided from the Laboratory of Retrovirology at Rockefeller University. Human embryonic kidney 293T (HEK 293T) were obtained from ATCC (REF CRL-3216). Dulbecco's Modified Eagle Medium (REF 31966021), Opti-MEM (REF 31985054), fetal bovine serum (REF 16140071), and Pen-Strep (REF 15070063) were obtained from Gibco. The transfection reagent, branched polyethylenimine 408727-250ML, was obtained from Sigma-Aldrich. The lysis buffer and the NanoGlo reagents for the luciferase assay were purchased from Promega, REF E1531 and REF N1150, respectively.

### 2.1. Peptide Synthesis

The ACE-2 peptide sequences were synthesized through solid-phase peptide synthesis (SPPS) by using a CS136X CS biopeptide synthesizer. MBHA rink amide resin was used for the solid support. In brief, TBTU (3 eq.), HOBt (3 eq.), DIPEA (6 eq.), and the desired Fmoc-protected amino acid (3 eq.) were used for each coupling reaction step. The Fmoc-protecting group was removed using a mixture of 20% (*v*/*v*) piperidine in DMF. After the completion of all the coupling reactions, the final peptides were cleaved from the resin by adding a mixture of 95% TFA, 2.5% TIS, and 2.5% water and stirred at room temperature for 2 h. The peptides were precipitated in diethyl ether and collected by centrifugation. The solids were dried under a vacuum overnight. Finally, the peptides were purified by a reverse-phase HPLC with a C-18 column. The peptides were precipitated in diethyl ether and collected by centrifugation. The solids were dried under a vacuum overnight. Finally, the peptides were purified by a reverse-phase HPLC with a C-18 column.

To calculate the purity of the peptides, we used liquid chromatography-mass spectrometry (LC-MS); Agilent 1260 Infinity LC; Agilent Zorbax SB-C18, 4.6 × 250 mm column; and Agilent 6130 Quadrupole MS. A 1.5 ml/min flow rate of a mobile phase was used in addition to 0.1% (*v*/*v*) formic acid with water (A) and 0.1% (*v*/*v*) formic acid with acetonitrile (B). The chromatogram was obtained at a wavelength of 220 nm in Figures S1A and S1B with a purity > 95%.

### 2.2. MST Binding Studies

The binding affinities of each ACE-2 peptide with the spike protein were examined through thermophoretic measurements taken with the red detection channel of the Monolith NT.115 instrument. The fluorescent dye NT647 was used to label the SARS-CoV-2 spike S1 to record MST following the protocol recommended by NanoTemper [22]. Here, 100 *μ*l NT647-N-hydroxysuccinimide fluorophore (NanoTemper Technologies) was incubated for 30 min in the dark at room temperature with 100 *μ*l of spike S1 protein in the labeling buffer (130 mM NaHCO_3_, 50 mM NaCl, and pH 8.2). After that, 10 *μ*l of 4.4 *μ*M NT647-Spike in MST buffer and 10 *μ*l of each peptide serial dilution in Tris buffer were mixed. This buffer can mimic the physiological pH conditions found in biological systems; this buffer can mimic the physiological pH conditions found in biological systems. In all the samples, the peptide's initial concentration was 500 *μ*M, and NT647-Spike's concentration was 5 nM. Following the loading of the samples into 16 premium-coated capillaries from NanoTemper Technologies, fluorescence was measured for 40 seconds while using 20% laser power and medium MST power. All measurements were conducted with the instrument's temperature set at 21°C. The data were examined following the recording of MST time traces. Using the ligand concentration-dependent variations in the normalized fluorescence, the *K*_D_ value was estimated. The provided values were produced using the MO Affinity Analysis software (NanoTemper Technologies).

### 2.3. ELISA Detection Studies

The ELISA experiments were conducted using a sandwich ELISA methodology. P1 and P25 peptides were dissolved in a carbonate-bicarbonate coating buffer to a final concentration of 10 *μ*M. Maleic anhydride-activated plates (Thermo Fisher, 15110) were coated with P1 and P25 peptides and incubated at 4°C overnight. ACE2 was used as a control, coated at a concentration of 1 *μ*g/ml. The plate wells were then washed with 1× PBS with 0.05% Tween-20 washing buffer, followed by the addition of a blocking buffer to each well (Thermo Fisher, N502), and then incubated overnight at 4°C. After repeating the washing step, 1 *μ*g/ml of the spike protein (Sino Biological, 40591-V08H) was added to the wells and incubated at room temperature for 1 h. This was followed by a washing cycle and then the addition of anti-spike antibodies (Invitrogen, PA5-81795) with a concentration of 1 *μ*g/ml. Following a 1 h incubation at room temperature and the washing cycles, a concentration of 1 *μ*g/ml of the secondary antibodies (Abcam, ab6721) was added and incubated for 1 h at room temperature. Then, the plates were washed and incubated with the TMB substrate (Abcam, ab171523) for 30 min at room temperature before adding the stopping solution (Invitrogen, SS04). The optical density (OD) signal was measured at 450 nm using a PHERAstar FS microplate reader.

### 2.4. Circular Dichroism Studies

The CD spectra were measured using an AVIV-430 spectrophotometer at scanning wavelengths between 190 and 300 nm, with 1.0 nm increments. The samples were placed in a 0.1 mm optical path length cuvette. Both the P1 and P25 peptides were dissolved at a concentration of 1 mg/ml in 1× PBS. The signals were normalized to the molar ellipticity value.

### 2.5. Cytotoxicity Assay

The cytotoxicity of the peptides was assessed using a viability assay. Modified HEK 293T cells expressing the ACE2 receptor (293T ACE-2 SSC22) were mixed with 100 *μ*l DMEM-supplemented media and incubated in 96-well plates with a seeding density of 2500 cells/well. After overnight incubation, the P1 and P25 peptides were added to the designated wells at an initial concentration of 4 mM, followed by subsequent 2-fold dilution until 0.125 mM was reached. Cells with peptides were cultured for 3 days, after which cell viability was measured using an ATP assay (Promega, G9681).

### 2.6. Molecular Docking Simulations

Two inhibition peptides were used as potential targets for the reported SARS-CoV-2 VOCs [23–27]. The analyzed variants were Alpha B.1.1.7, Beta B.1.351, Gamma P.1, Delta B.1.617.2, Epsilon B.1.429, and Omicron B.1.1.529 (PDB codes: 7LWV, 7LYN, 7SBO, 7M8K, 7N8H, and 7T9J). In addition, the spike mutation D614G, which is associated with increased infectivity, was evaluated (PDB: 7BNN) [28]. To obtain an accurate initial three-dimensional conformation of these peptides, the protein folding prediction approach (AlphaFold) was used to determine their configuration [29, 30]. The AlphaFold prediction was run in single-sequence mode.

Then, docking simulations between the peptides and SARS-CoV-2 variants were conducted using the ClusPro protein-protein docking methodology [31–35]. The receptor to ligand method was ran using ClusPro to compute the molecular docking simulations. The top simulations were generated through ClusPro according to their cluster, members, center, and lowest energy-weighted scores. A positive control was developed by verifying the docking between the previously reported inhibition peptides and the original SARS-CoV-2 (PDB code: 6M0J) receptor [21, 36]. Next, the top ten simulations of each docking were analyzed considering their rank and through their energy scores after *Z*-score normalization. The *Z*-score normalization was performed according to Equation (3). The polar contact interactions were visualized and analyzed using PyMOL (v4.6.0). The main interactions between the peptides and the SARS-CoV-2 variants considered to evaluate the effectiveness of the peptides were those pertaining to the reported RBD domain from the spike protein [36, 37]. Subsequently, to compare P1 and P25 binding against the SARS-CoV-2 strains, a comparative fold between these peptides was calculated considering only the RBD interactions using Equation (1). On the other hand, Equation (2) was used to separately evaluate each peptide amino acid sequence interaction with the SARS-CoV-2 variants.

(1) $$\begin{array}{c} \text{Comparative interaction fold}=\left(\frac{\alpha }{\beta }\right). \end{array}$$

Equation (1) is the comparison between P25 and P1 interaction's fold, where *α* represents the total interactions from P25 against the SARS-CoV-2 and variants, while *β* represent the total interactions from peptide inhibitor P1 against the SARS-CoV-2 and variants, respectively.

(2) $$\begin{array}{c} \text{Relative interactions from peptide composition}=\left(\frac{\chi *100}{\xi }\right). \end{array}$$

Equation (2) is the relative interactions from peptide composition, where *χ* represents the total interactions for a given amino acid within the peptide composition and *ξ* represents the total amino acid interactions from the entire peptide. The result is expressed in terms of percentage.

(3) $$\begin{array}{c} Z=\left(\frac{x-\mu }{\sigma }\right). \end{array}$$

Equation (3) is the *Z*-score normalization, where *Z* is the calculated *Z*-score value, *x* is the value to be normalized, *μ* represents the mean, and *σ* is the standard deviation.

### 2.7. Plasmids, Cell Culture, and Pseudovirus Production

The study used HEK 293T cells to produce a replication-defective HIV-1-based SARS-CoV-2-pseudotyped virus. The pseudovirus was produced similarly to the commonly used approach of lentivirus vector production, which usually consists of a three-plasmid system. The first plasmid contained a packageable reporter gene encoding GFP and a NanoLuc reporter gene, which was used for the titration assay. The packaging plasmid, which is composed of an HIV-1 backbone, contained structural and regulatory genes, including gag and pol. The third plasmid contained a truncated and codon-optimized SARS-CoV-2 spike gene. The Omicron and Delta spike gene sequences were obtained by modifying the original SARS-CoV-2 spike protein sequence. The plasmid containing the original sequence was modified to include all reported mutations of each variant [38]. After codon optimization, the targeted sequence was then subcloned into the desired plasmid by restriction digestion using PCiI (Cat: R0655S) and SacI-HF (Cat: R3156S) and confirmed by the Sanger sequencer using the primers highlighted in Table S1.

The pseudovirus production protocol was adapted from Schmidt et al. with some modifications [39]. In brief, HEK 293T cells with 10% FBS and 1% Pen-Strep were cultured in DMEM until they reached 80% confluency. On the first day of the production, 5 million HEK 293T cells were incubated in a 100 mm dish overnight. Then, 24 hours following the cell seeding, the media was replaced with Opti-MEM combined with 10% FBS and 1% Pen-Strep. The three plasmids (reporter, packaging, and spike) were mixed with the optimum ratios (14 *μ*g : 14 *μ*g : 5 *μ*g) in serum-free Opti-MEM media. Then, 132 *μ*l of the transfection reagent, branched PEI, was diluted separately in serum-free Opti-MEM to a working concentration of 1 mg/ml. The two suspensions were then mixed and incubated for 20 minutes at room temperature. After incubation, the mixture was added to the seeded cells. After 48 hours, supernatants were collected, filtered, concentrated, and titered to obtain the TCID50, which was calculated according to the Reed-Muench method by using the “TCID50_SARS-CoV-2” macro provided by Nie et al. [40].

### 2.8. TEM Characterization

The assembly of the pseudovirus was evaluated using a field emission gun Tecnai Twin 300 kV transmission electron microscope (TEM). The sample was prepared by adding one drop onto a TEM copper grid and incubating at room temperature for 3 minutes. The excess solution was removed, and then, the grid was stained with a drop of 2% uranyl acetate for 30 seconds. The grid was then dried at room temperature overnight.

### 2.9. Pseudovirus Titration

The pseudovirus was titered to standardize viral infection. Briefly, HEK 293T cells expressing the ACE2 receptor (293T ACE-2 SSC22) were seeded into a 96-well plate with 10,000 cells/well in 100 *μ*l of DMEM-supplemented media. After reaching confluency, a twofold dilution of the pseudovirus was added to each designated well and incubated for 48 hours. After the incubation, the cells were lysed and the luminescence of luciferase was measured using a PHERAstar FS, BMG LABTECH luminometer reader.

### 2.10. Neutralization Assay

The efficiency of peptide inhibition was tested using a neutralization assay. Here, 293T ACE-2 SSC22 cells were seeded into a 96-well plate with 10,000 cells/well in 100 *μ*l DMEM-supplemented media. The lyophilized peptides were dissolved in a carbonate-bicarbonate buffer to an initial concentration of 4.0 mM. Using a twofold dilution factor, different concentrations of the peptides were tested for their inhibitory effect with a constant viral titer of 13,000 TCID50/ml of the original or VOCs of pseudovirus. As for the controls, each pseudovirus was directly used to infect the target cells without peptide pretreatment. As for the experimental group, 50 *μ*l of the dissolved peptides was incubated with equal volumes of pseudovirus suspension for 1 hour at 37°C. After incubation, the mixed samples were used to infect previously seeded cells. After 48 hours of incubation, the samples were washed once with 1× PBS and then treated with 50 *μ*l of 1× lysis buffer for 15 minutes at room temperature. The luciferase assay was carried out in a black-walled 96-well plate by mixing equal volumes of the cell lysate and the NanoGlo reagents. The luciferase luminescence was performed using a PHERAstar FS, BMG LABTECH luminometer reader. The results of the assay were analyzed by comparing the luminescence or fluorescence signal from the cells that were incubated with inhibitory peptides to the signal from the cells that were not incubated with inhibitory peptides. The lower the luminescence or fluorescence signal, the more effectively the inhibitory peptides have neutralized the virus.

## 3. Results and Discussion

### 3.1. Peptide Synthesis

The two selected peptides were synthesized using solid-phase peptide synthesis (SPPS). The resultant peptides were P1 and, a mutated sequence from the same region, P25, as shown in Table 1. The peptides' molecular weight and purity were calculated using mass spectroscopy and high-performance liquid chromatography. All peptides were >95% pure. Each run showed a peak of relative abundance on the *y*-axis and mass over the peptide charge on the *x*-axis for P1 and P25, as shown in the supplementary Figures S1A and S1B.

### 3.2. Binding Studies

**Figure 2:**
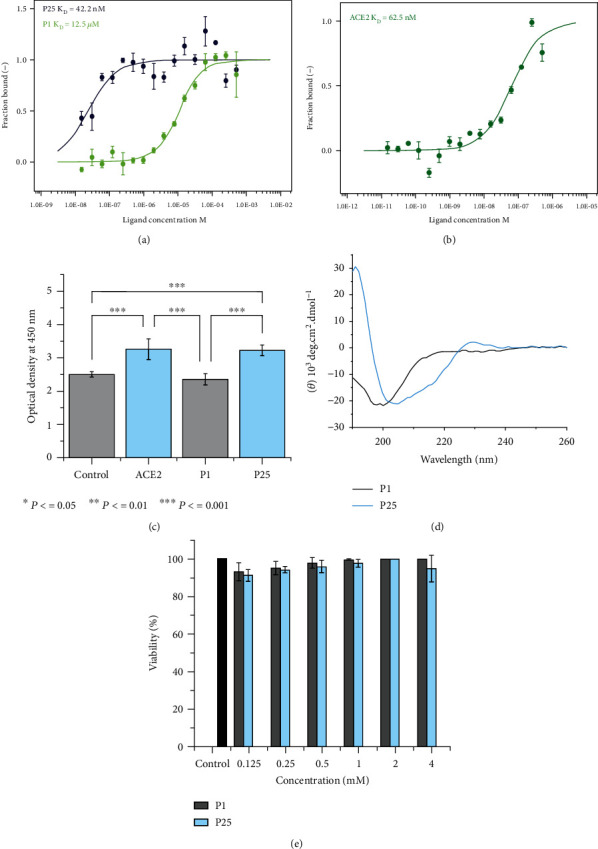
MST binding analysis between the spike S1 protein and P25 peptide, P1 peptide (a), and ACE2 as a positive control (b). (c) The ELISA OD signals for P1 and P25 peptides from a sandwich ELISA indicate stronger binding between the P25 peptide and spike protein (*n* = 3). (d) The CD spectra of P1 and P25 in 1× phosphate-buffered saline (PBS) at a concentration of 1 mg/ml. (e) 293T ACE-2 SSC22 cell viability after testing with P1 and P25 peptides at different concentrations for 72 hours. Error bars represent SD (*n* = 3).

The binding affinity of each peptide with the SARS-CoV-2 spike protein was evaluated by MicroScale Thermophoresis (MST) and ELISA assays. MST is a technique that analyzes the interaction between biomolecules by detecting the temperature-induced change in the fluorescence of a target. The constants of dissociation (*K*_D_) for P25 and P1 were found to be 42.2 nM and 12.5 *μ*M, respectively (Figure 2(a)). A lower *K*_D_ value signifies better affinity and stronger interaction. The binding affinity was also tested for ACE2 with the spike S protein and found to be 62.5 nM, as indicated in Figure 2(b). The *K*_D_ of P25 was comparable to the observed affinity between the S protein and ACE2. Similarly, in ELISA, the reported results in Figure 2(c) showed a higher optical density (OD) signal for P25 in comparison to P1 and negative control (*p* value < 0.001. The results were similar between P25 peptide and ACE2 control. These results were consistent with the previous study [41].

### 3.3. Circular Dichroism Spectrophotometer Analysis

The secondary structure of the synthesized peptide was assessed using circular dichroism (CD).  Figure 2(d) illustrates the CD spectra of P1 and P25. The P1 peptide exhibited a random coil conformation. A transitional change in the confirmation from random coil to alpha-helix was observed for P25. P1 had spectra with a minimum negative peak at 200 nm, which is a characteristic of a random coil structure. In contrast, P25 had a negative minimum peak at 205, which indicates a shift toward a more alpha-helical structure. We believe that this conformational change contributes to the increased binding affinity of P25, as observed in the MST.

### 3.4. Cytotoxicity

A cytotoxicity test was performed on HEK 293T cells expressing ACE2 receptor (293T ACE-2 SSC22), as shown in Figure 2(e) and Supplemental Figure S2. The tested peptides did not show cytotoxic effects at the tested concentration, with cell viability ranging between 91 and 100% after 72 hours of treatment. The low toxicity exhibited by P25 and P1 indicates that these peptides are biocompatible, even at high concentrations (e.g., at 4 mM). Cytotoxicity data for the ACE2 protein is provided in the supplemental Figure S3.

### 3.5. *In Silico* Evaluation: Molecular Docking

**Figure 3:**
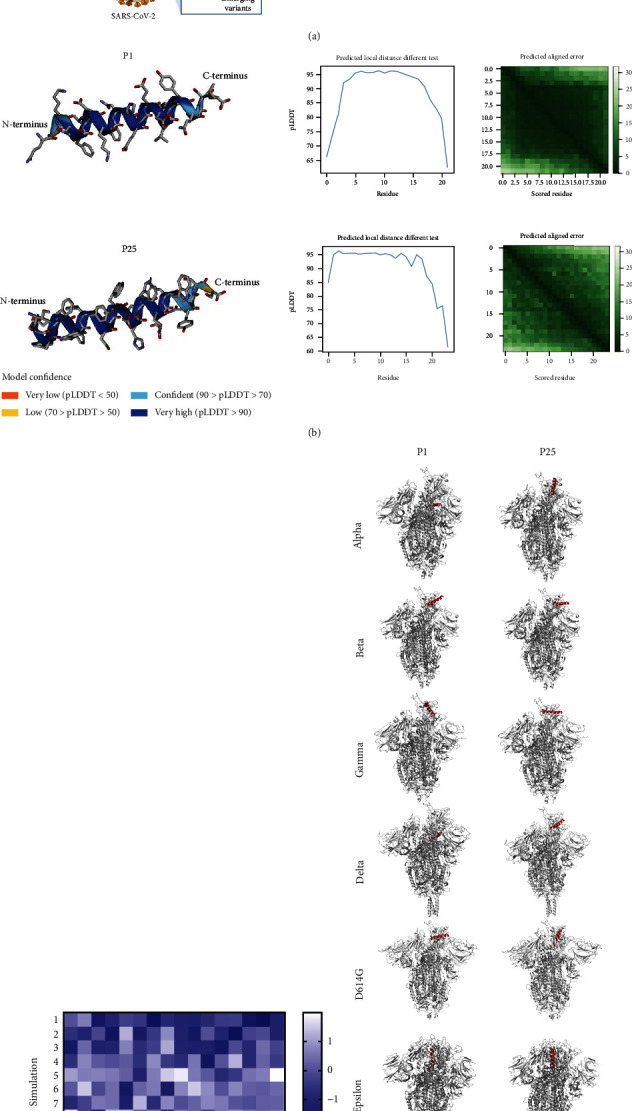
Overview of the molecular docking simulations (a). The peptide 1 (top) and peptide 25 (down) tridimensional structural predictions from AlphaFold, predicted confidence (pLDDT), and predicted aligned error (PAE) are shown (b). (c) The top 10 simulation *Z*-scores representing the molecular docking between the peptides and the virus variants of concern. The top molecular docking simulations between the peptides to the SARS-CoV-2 variants and the spike mutation D614G are shown (d).

The peptides exhibited several interactions with the RBD region of the SARS-CoV-2 virus and its variants. An overview of the approach followed for the molecular docking simulations is shown in Figure 3(a). As an initial control, the binding results from the de novo folded peptide with AlphaFold were compared against the reported binding study from Panda et al. [21], eight interactions were observed in comparison to the original SARS-CoV-2 strain, and seven interactions were observed for P25 (Table S2). These results support the interaction of P1 and P25 with the spike protein while also remaining congruent with the molecular docking study of the original SARS-CoV-2 strain from Panda et al. [21]. *In silico*, the peptides' structure prediction by AlphaFold showed that both P1 and P25 fold mainly into helix structures (Figure 3(b)). However, P1 exhibit a random coil folding in the CD experiment which indicate an influence of external factors. To compare against our experimental results, simulations were carried for P1 and P25 against the VOCs: Delta and Omicron. Additionally, the molecular docking between the peptides and variants of concern (Alpha, Beta, Gamma, and Epsilon) and the presence of the mutation D614G were evaluated and reported at the supporting information (Table S2 and Figures S4 to S9); we utilized these data to later introduce in this paper an optimization strategy for the future design of inhibitory peptides against emerging SARS-CoV-2 variants.

Next, the top 10 simulation results from ClusPro were evaluated according to their lowest energy scores as shown in Figure 3(c) and Supplementary Figure S10. We observed that these peptides could interact with several emerging SARS-CoV-2 VOCs (Figure 3(d)). In these simulations, P25 exhibited 1.82 times more interactions than P1. This fold was calculated according to Equation (1). Furthermore, P25 exhibited interactions within the RBD of all variants, while P1 did not show any interaction within the RBD against the Delta variant. P1 exhibited several interactions outside of the RBD region (Table S2), which suggest that this peptide is less specific in targeting this region against the variants. This could be due to the presence of mutations within the SARS-CoV-2 variants [42, 43].  Table 2 shows the main interactions between both peptides and the original SARS-CoV-2 strain. It also shows the interactions between the two peptides and the Delta and Omicron variants.

According to our results, P25 exhibited 1.25 times more interactions against the Omicron variant in comparison to P1 (Table 2) and 1.4 times more interactions than P1 against the D614G mutation (Table S2). Further, P25 shared key binding sites against the RBD (TYR 449, ASN 487, TYR 489, and GLN 493) that have been reported from highly potential inhibitors against the S-RBD of the SARS-CoV-2 [44].

**Figure 4:**
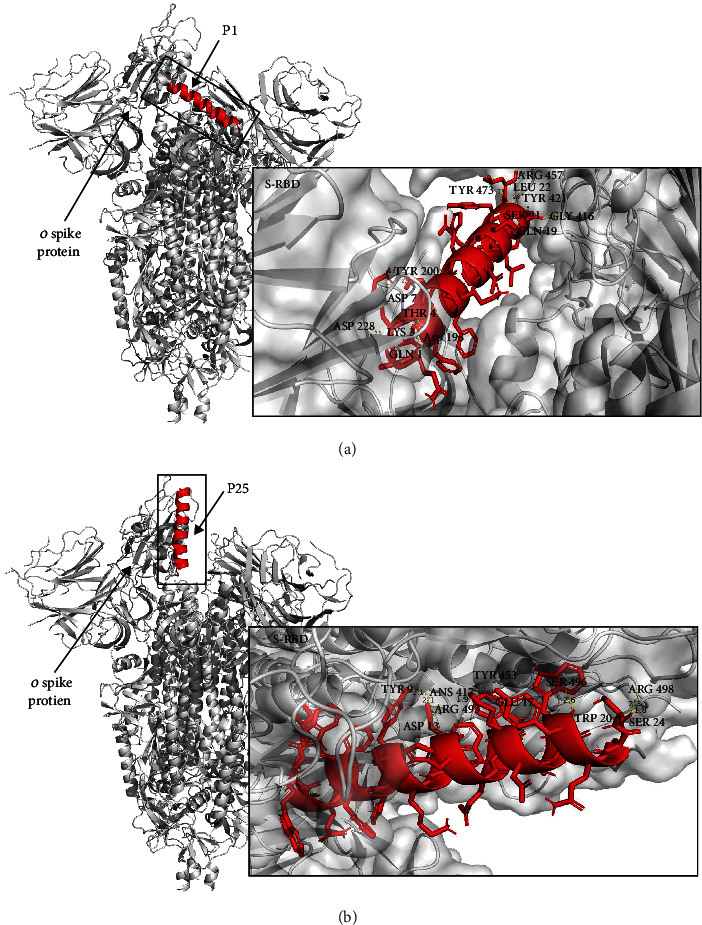
Molecular docking between peptide inhibitors P1 (a) and P25 (b) with the Omicron spike protein.

In particular, for Omicron, it was found that P25 interacts with several of the reported mutations occurring within the S-RBD of the Omicron variant (Figure 4) [45]. For Omicron, these interactions included ASN 417, ARG 493, SER 496, and ARG 498. In contrast, P1 did not exhibit interactions with any of the reported S-RBD mutations (Table 2). The mutations at residues 493, 496, and 498 have been reported to contribute significantly to the binding affinity from Omicron to the ACE2 receptor [46]. In particular, residues such as the mutation 493 increase binding affinity and result in a potential challenge for predicting transmissibility and immune evasion risk [43]. Therefore, by interacting with these amino acids, P25 might exhibit better properties to act as an inhibitor for Omicron than P1. Furthermore, P25 interacted with the residue LYS 417 from the original SARS-CoV-2 strain while also interacting with the same residue that is conserved in Delta and its mutation K417N that is present in Omicron, which suggests that P25 might be more effective at targeting the RBD than P1. These findings highlight the relevance of P25's amino acid composition in relation to P25's ability to interact with the original virus strain and the Omicron variant, paving the way for furthering optimization of these peptide sequences. However, further experiments could be done to verify experimentally these interactions.

### 3.6. Production of the Pseudovirus and the Variants

**Figure 5:**
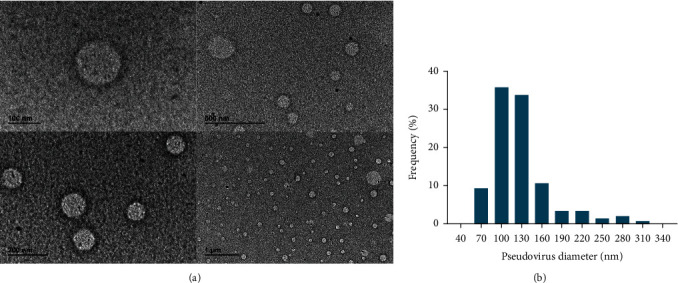
(a) TEM image of assembled pseudovirus. (b) Size distribution of pseudovirus in percentage.

Due to the high pathogenicity of the virus, we used a pseudotyped virus, which offers significant advantages over using a live virus, such as the pseudovirus's versatility and safe handling. The size and shape of the produced pseudovirus were evaluated by transmission electron microscope (TEM) images in Figure 5(a). Over 500 particles of the assembled pseudoviruses were analyzed using ImageJ and GraphPad to determine their frequency size distribution. The assembled pseudoviruses exhibited variations in size, with approximately 70% of them ranging between 85 and 145 nm in diameter (see Figure 5(b)). The functionality of the produced pseudovirus was tested against HEK 293T cells expressing ACE2 receptor by the detection of the green fluorescence protein signal (GFP) (Figure S11 A-C) and the measurement of luciferase luminescence from infected cells. These results confirmed that the produced pseudovirus is functional. Furthermore, the specificity of the SARS-CoV-2 pseudovirus for the ACE2 receptor was confirmed by testing it against HEK293T wild-type cells (Figure S11D).

### 3.7. Neutralization of SARS-CoV-2 and Variant Pseudoviruses

**Figure 6:**
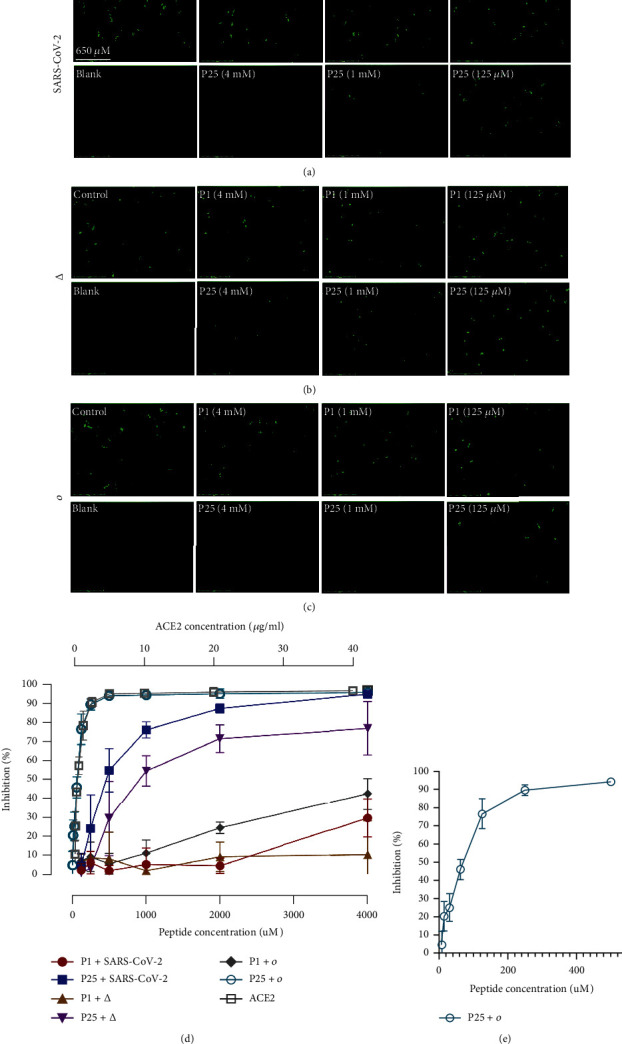
(a–c) Fluorescent microscope images of GFP-positive cells after 48 h of SARS-CoV-2, Delta, and Omicron pseudovirus infection. The control contains pseudovirus without peptides and the blank contains cells only. Scale bar equals 650 *μ*m (*n* = 3). (d) Neutralization assay of P1 and P25 peptide inhibition of pseudovirus variants (*n* = 3). (e) P25 inhibition of Omicron pseudovirus at lower concentrations (*n* = 3).

We evaluated the inhibitory activity of two potential binding inhibitor peptides, P1 and P25, against the original SARS-CoV-2 pseudovirus strain and the Delta and Omicron variants. In addition, we tested the peptides against vesicular stomatitis virus (VSV) pseudovirus to analyze their specificity (Figure S12). The inhibitory activity of each peptide as well as ACE2 protein against pseudovirus was evaluated by the reduction in the luminescence signal measurement (Figure 6(d)) and the overall number of GFP-positive cells as observed under a fluorescent microscope (Figures 6(a)–6(c)). P25 exhibited a strong inhibition of pseudovirus entry in a dose-dependent manner. The number of infected cells gradually increased with the reduction of peptide concentration from 4 mM to 125 *μ*M, as demonstrated by the increase in GFP signal, which indicated that the P25 peptide was the cause of the prevention of viral entry (Figures 6(a)–6(c)). Although the inhibition potency of P25 was weaker with the Delta variant in comparison to the original virus (Figure 6(b)), P25 showed inhibition potency against the Omicron variant even at a low peptide concentration (Figure 6(e)). The calculated IC_50_ for P25 against the original SARS-CoV-2 strain, the Delta variant, and the Omicron variant was 455.2 *μ*M, 546.4 *μ*M, and 60.8 *μ*M, respectively. The significantly low IC_50_ for P25 against the Omicron variant makes this peptide a promising potential candidate against Omicron subvariants.

The increased inhibitory activity of P25 against the Omicron variant might be attributed to both P25's high negative charge and Omicron's high positive charge in comparison to the original strain and the other variants [47]. Moreover, the CD spectrum analysis indicated a shift to alpha-helical content in the P25 peptide, potentially contributing to the stronger inhibition observed in our neutralization assay. However, when compared to the previously reported peptides, P25 remained less potent than some fusion inhibitors that had only been tested against the original strain [18]. Nevertheless, we believe that the potency of P25 can be improved through functionalization and further optimization of the peptide sequence.

P1 showed limited inhibition at the highest concentration: 4.0 mM. We believe that the lower inhibitory effect of P1 is attributed to the random coil secondary structure observed in Figure 2(e) of the CD analysis. Moreover, our simulation suggested a lower specificity for P1, which is represented by the number of interactions between P1 and the spike residues located outside the RBD. Hence, another factor contributed to the decreased P1 inhibition. Generally, the experimental results were aligned with our simulation, which demonstrated that P25 was a better inhibitor against the SARS-CoV-2 VOCs as supported by the overall secondary conformation and number of interactions with the RBD region.

### 3.8. *In Silico* Evaluation: Peptide Design and Potential Optimization

**Figure 7:**
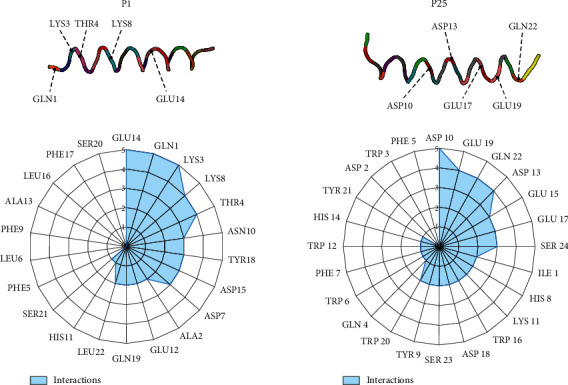
Distribution of the main interacting amino acids against the SARS-CoV-2 variants from the peptide composition.

We further analyzed the peptide inhibitors and determined the main amino acids from their composition that interacted against the SARS-CoV-2 and the Alpha, Beta, Delta, Gamma, Epsilon, and Omicron variants (Figures S4-S9 and Table S2) shown in Figure 7. The contribution of each amino acid was calculated using Equation (2). Considering the overall amino acid composition of P25, it was found that 87.5% of its amino acids interact with the RBD; this was calculated considering if the amino acid from peptide exhibited at least one binding against the original SARS-CoV-2 and variants (see Equation (2)). On the other hand, for P1, only 68.2% of its sequence composition was interacting with the RBD. We concluded from the number of amino acids interacting with the SARS-CoV-2 variants that P25 displayed a better amino acid composition for interacting with the RBD domain than P1. In addition, since P25 exhibited the most interactions, it could potentially be modified to generate a stronger interacting peptide against emerging variants. This could be done by reserving the most interacting amino acids from the inhibitor peptide sequence and then removing or substituting the noninteracting amino acids from the peptide. According to our computational simulations, the main interacting amino acids from P25 against the variants were ASP 10, ASP 13, GLU 17, GLU 19, and GLN 22 (Figure 7). Hence, this peptide could be potentially trimmed and redesigned while maintaining these main interacting amino acids in its sequence. This approach could be used as a mitigation strategy for the quick design of peptide-based inhibitors against emerging viruses.

## 4. Conclusion

We reported on the experimental synthesis and evaluation of two potential binding inhibitor peptides, P1 and P25, against the SARS-CoV-2 VOCs. Our molecular docking simulations demonstrate that both peptides interact with different SARS-CoV-2 variants. However, the amino acid composition design and structure of P25 enable more specific targeting of the RBD than P1. In agreement with the *in silico* analysis, our binding study suggested a high affinity of P25 to the spike protein and can inhibit the entry of the virus into cells, which was also comparable to the binding affinity of the full-length ACE2 protein to the spike protein. This inhibition was observed in vitro against the pseudovirus of the original SARS-CoV-2 strain, the Delta variant, and the Omicron variant. Nevertheless, further studies are needed to assess the efficacy and potency of P25 in vivo. These studies should include animal models and clinical trials.

We believe that further modification and optimization of the peptide could be exploited to improve P25's efficacy and potency against emerging variants. However, it is important to note that the therapeutic benefits of these peptides have not yet been established. More research is needed to determine whether P25 can be used as an effective antiviral drug to treat COVID-19.

## 5. Statistical Analysis

GraphPad Prism software program (GraphPad Software, La Jolla, CA) was used to calculate the IC_50_ of the peptides and the TCID50 of each pseudovirus type.

## Figures and Tables

**Table 1:** Sequences of ACE2-derived peptides.

Peptide	Position	Region	Sequence
P1	24-45	ACE2 receptor	QAKTFLDKFNHEAEDLFYQSSL-amide
P25	21-44	ACE2 mutated	IDWQFWFHYDKWDHEWEDEWYQSS-amide

**Table 2:** Summary of the interactions from the spike of the original SARS-CoV-2, Delta, and Omicron variants against the designed peptides.

Peptide	Spike origin	RBD conformational state	Interactions within RBD	Interactions outside the RBD	Interactions from peptide
P1	SARS-CoV-2	1 RBD-up	ARG 403, LYS 417, TYR 453, GLN 493, GLN 498, THR 500, ASN 501, TYR 505	—	GLN 1, LYS 3, THR 4, ASP 7, ASN 10, GLU 14, TYR 18
	Delta	1 RBD-up	—	LYS 41, ASP 198, TYR 200, ASP 228, GLN 755, ILE 973, ARG 983	GLN 1, ALA 2, LYS 3, LYS 8, HIS 11, GLU 12, GLU 14
	Omicron	1 RBD-up	GLY 416, TYR 421, ARG 457, TYR 473	ASP 198, TYR 200, ASP 228	GLN 1, LYS 3, THR 4, ASP 7, GLN 19, SER 21, LEU 22

P25	SARS-CoV-2	1 RBD-up	ARG 403, LYS 417, ASP 420, TYR 449, GLN 493, ASN 460, TYR 505	—	ILE 1, TRP 6, ASP 10, ASP 13, GLU 17, TYR 21
	Delta	1 RBD-up	ARG 403, LYS 417, TYR 453, ASN 487, TYR 489, GLN 493	—	ASP 10, HIS 14, GLU 19, GLN 22, SER 23
	Omicron	1 RBD-up	ASN 417, TYR 453, ARG 493, SER 496, ARG 498	—	TYR 9, ASP 13, GLU 17, TRP 20, SER 24

## Data Availability

The data used to support the findings of this study are available from the corresponding author upon request.

## Abbreviations

ELISA: Enzyme-linked immunosorbent assay
FBS: Fetal bovine serum
PBS: Phosphate-buffered saline
DMEM: Dulbecco's Modified Eagle Medium
TCID50: 50% tissue culture infectious dose
HEK: Human embryonic kidney
PEI: Polyethylenimine
HIV: Human immunodeficiency virus
TMB: 3,3′,5,5′-Tetramethylbenzidine.

## Supplementary Materials

Supplementary Materials The Supporting Information is available free of charge on the ACS Publications website. Figure S1: (A, B) liquid chromatogram (LC-MS) at 220 nm and mass spectrum for P1 (A) and for P25 (B). Figure S2: cell viability assay with P1 and P25 peptides. Figure S3: cell viability assay with ACE2 protein peptides. Figures S4-S9: molecular docking of peptides and SARS-CoV-2 variants. Figure S10: scatter central cluster analysis. Figure S11: (A–D) GFP of pseudovirus production. Figure S12: neutralization assay of peptides against VSV pseudovirus. Table S1: list of primers for sequencing confirmation of Delta and Omicron pseudovirus variants. Table S2: comparative summary of the main interactions from the spike of the original and variants of concern.
