# Supplementary material for: Evaluation of Potential Peptide-Based Inhibitors against SARS-CoV-2 and Variants of Concern
Source: Biomed Res Int. 2023 Oct 13;2023:3892370. doi: 10.1155/2023/3892370 (PMC10589072; doi:10.1155/2023/3892370)
Supplement: Supplementary Materials — The Supporting Information is available free of charge on the ACS Publications website. Figure S1: (A, B) liquid chromatogram (LC-MS) at 220 nm and mass spectrum for P1 (A) and for P25 (B). Figure S2: cell viability assay with P1 and P25 peptides. Figure S3: cell viability assay with ACE2 protein peptides. Figures S4-S9: molecular docking of peptides and SARS-CoV-2 variants. Figure S10: scatter central cluster analysis. Figure S11: (A–D) GFP of pseudovirus production. Figure S12: neutralization assay of peptides against VSV pseudovirus. Table S1: list of primers for sequencing confirmation of Delta and Omicron pseudovirus variants. Table S2: comparative summary of the main interactions from the spike of the original and variants of concern. [file 3892370.f1.docx]

Evaluation of Potential Peptide-Based Inhibitors Against SARS-CoV-2 and Variants of Concern

**Supporting Information**

Hattan Boshah^1,2#^, Faris Samkari^1,2#^, Alexander U. Valle-Pérez^1,2^, Sarah M. Alsawaf^1,2^, Ali H. Aldoukhi^1,2^, Panayiotis Bilalis^1,2^, Salwa A. Alshehri^1,3^, H. H. Susapto^1^, and Charlotte A.E. Hauser^1,2*^

^1^ Laboratory for Nanomedicine, Division of Biological and Environmental Science and Engineering, King Abdullah University of Science and Technology, 23955 Thuwal, Kingdom of Saudi Arabia

^2^ Computational Bioscience Research Center, King Abdullah University of Science and Technology, Thuwal 23955-6900, Kingdom of Saudi Arabia

^3^ Department of Biochemistry, Faculty of Science, University of Jeddah, Jeddah, Saudi Arabia

^#^ These authors contributed equally

*Corresponding Author

**List of contents:**

**Figure S1(A, B)** Liquid chromatogram (LC-MS) at 220 nm and mass spectrum……….……….2

**Figure S2** Cell viability assay with P1 and P25 peptides…………….……………...……………2 **Figure S3** Cell viability assay with ACE2 protein peptides………..………………..……………3

**Figure S4 to S9** Show molecular docking of peptides and SARS-CoV-2 variants…………….3-6

**Figure S10** Scatter Central clusters analysis ………………………………..………………………6

**Figure S11(A-C)** GFP of pseudovirus production……………………………..…………………7

**Figure S12.** Neutralization assay of peptides against (VSV) pseudovirus……………….……………7

**Table S1** List of sequencing primers for pseudovirus variants…………………………………...8

**Table S2** Comparative summary of the main interactions from the spike protein……..……..9-10





**Figure S1.** Liquid chromatogram (LC-MS) at 220 nm and mass spectrum for P1 (A); and for P25 (B). The MS for P1 (m/z) calculated 2630.9, [M+3H]^3+^ found 871.8, [M+4H]^4+^ found 658.4 and [M+2H]^2+^ found 1307.1 (A); and for P25 the MS (m/z) calculated 3362, [M+4H]^4+^ found 841.5, and [M+3H]^3+^ found 1121.6, and [M+5H]^5+^ found 673.4(B).





**Figure S2:** 293T ACE-2 SSC22 cell viability assay after testing with P1 and P25 peptides at

different concentrations for 72 hours.





**Figure S3:** 293T ACE-2 SSC22 cell viability assay after testing with ACE2 protein at

different concentrations for 72 hours.

**
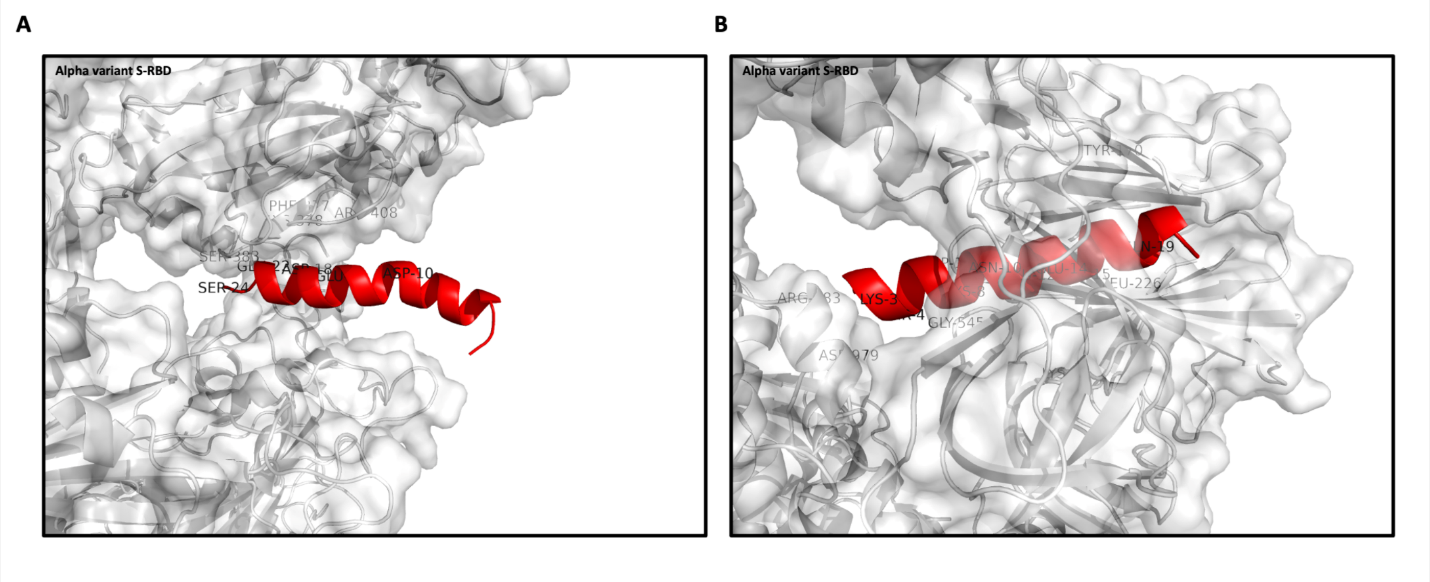
Figure S4**. Molecular docking between the peptide inhibitors P25 (A) and P1 (B) with the Alpha variant spike RBD.

**
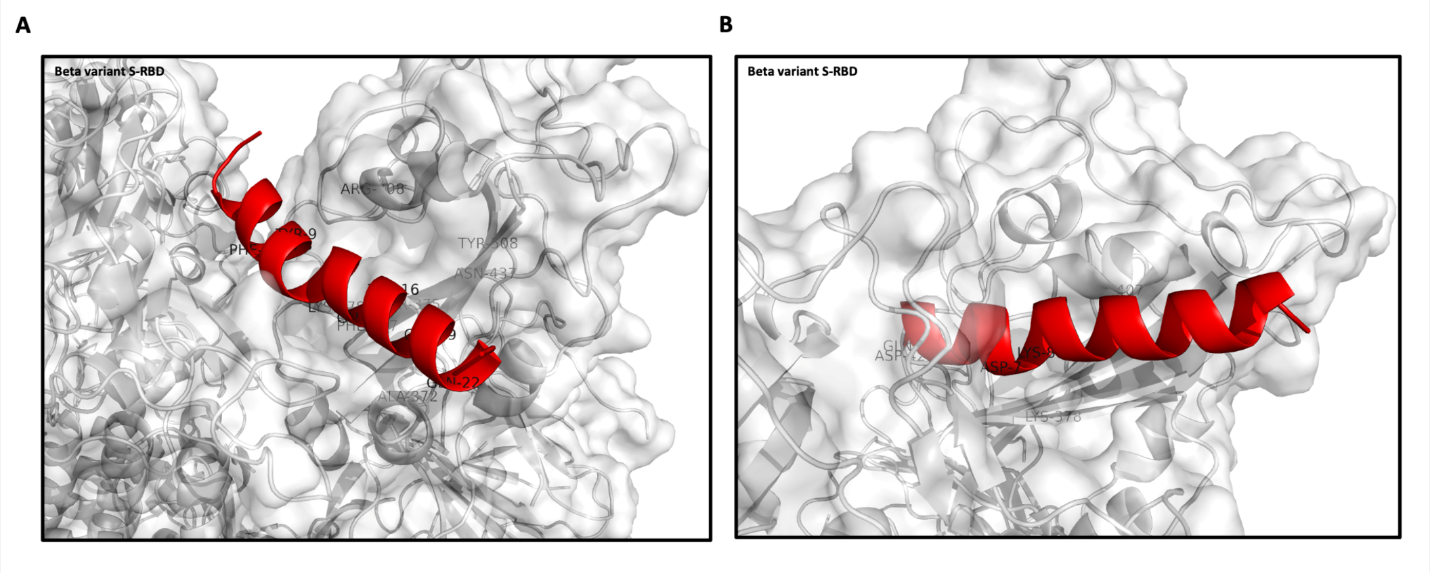
Figure S5**. Molecular docking between the peptide inhibitors P25 (A) and P1 (B) with the Beta variant spike RBD.


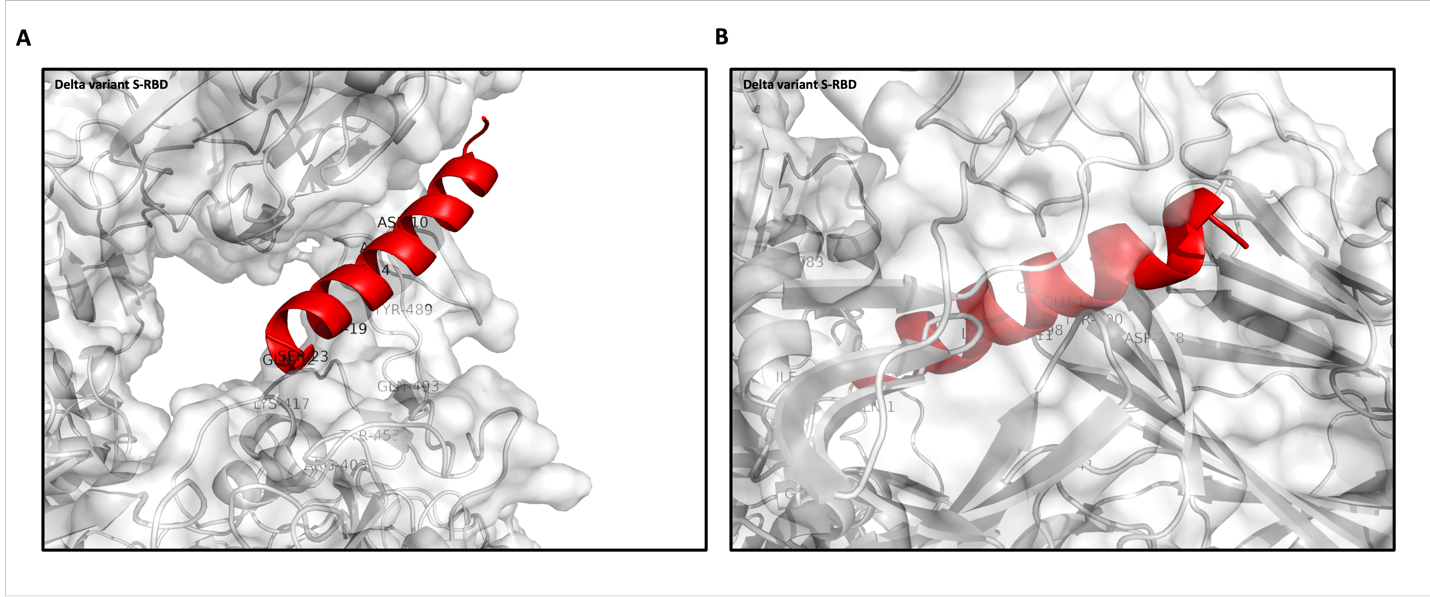


**Figure S6**. Molecular docking between the peptide inhibitors P25 (A) and P1 (B) with the Delta variant spike RBD.

**
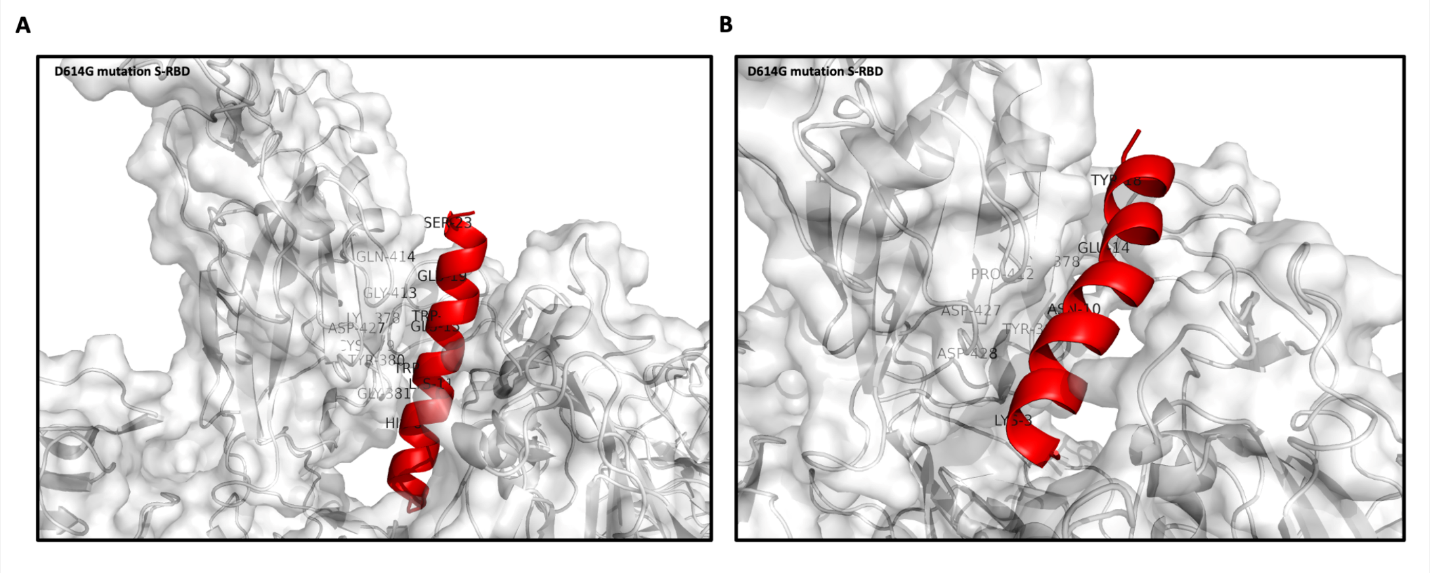
**

**Figure S7**. Molecular docking between the peptide inhibitors P25 (A) and P1 (B) with the D614G mutation spike RBD.


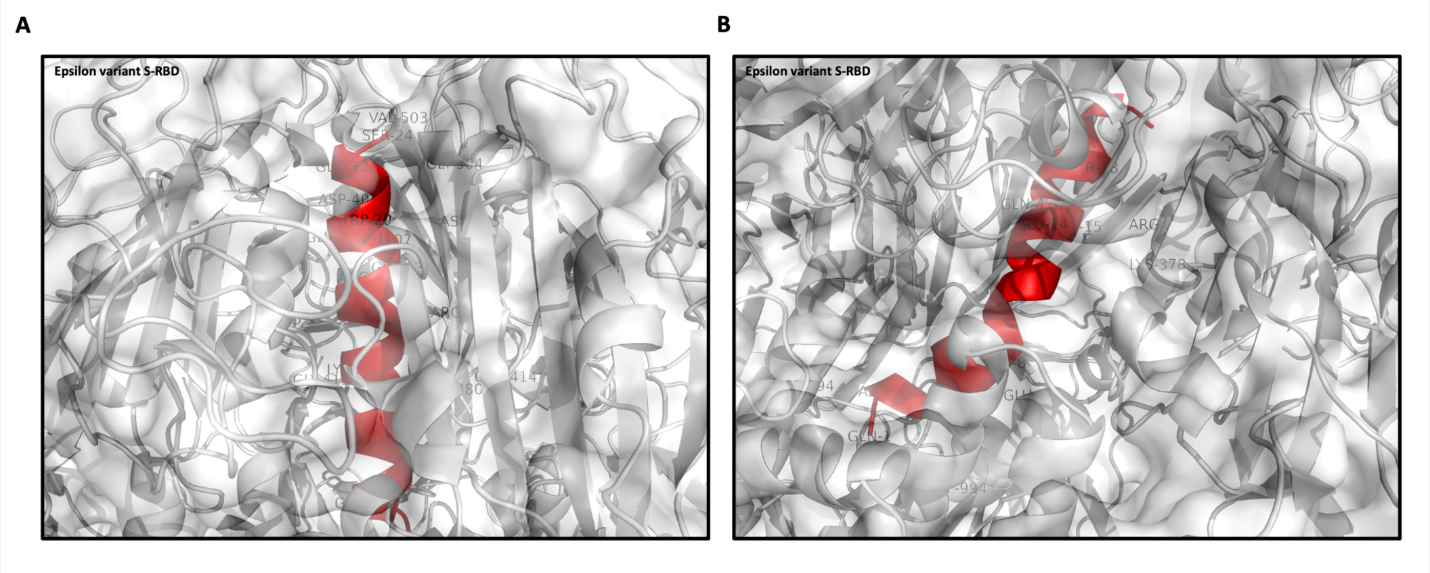


**Figure S8**. Molecular docking between the peptide inhibitors P25 (A) and P1 (B) with the Epsilon variant spike RBD.


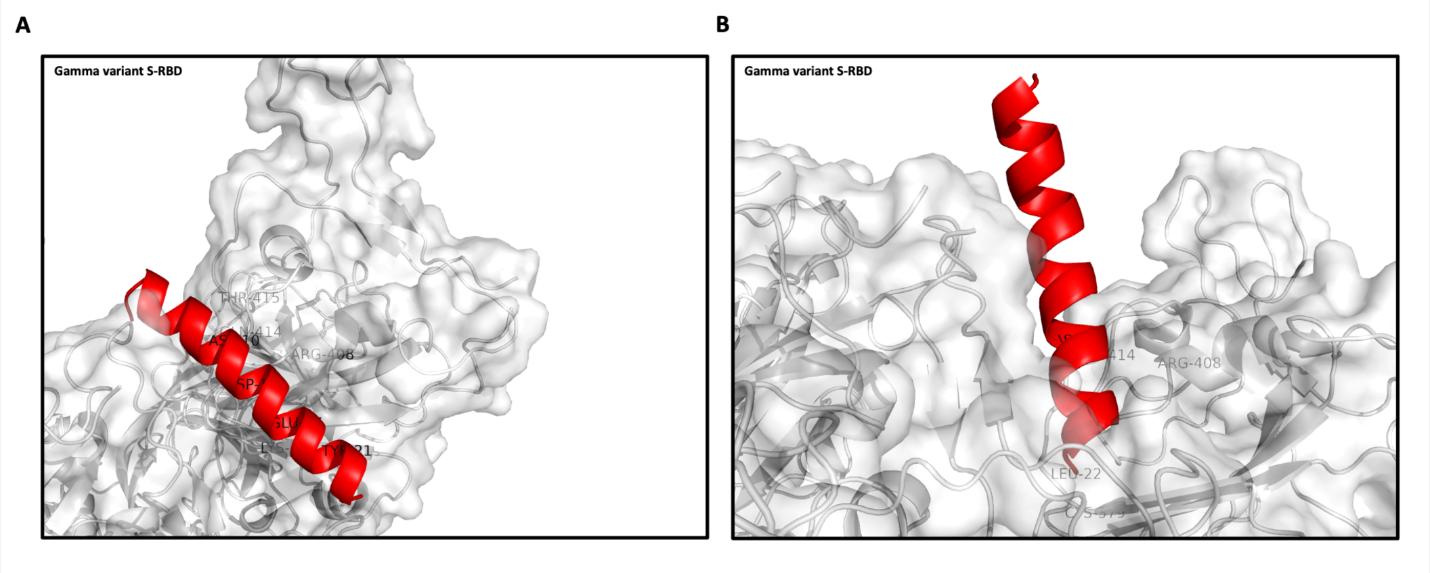


**Figure S9**. Molecular docking between the peptide inhibitors P25 (A) and P1 (B) with the Gamma variant spike RBD.


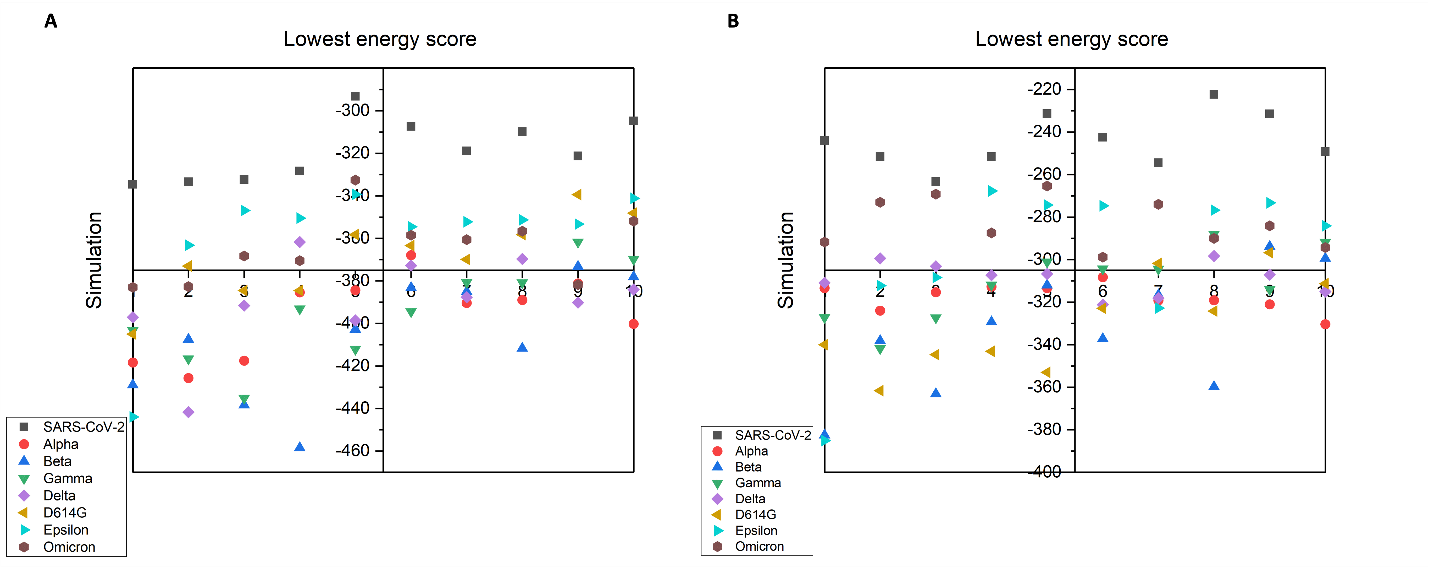


**Figure S10**. Scatter Central clusters analysis for the molecular docking simulation with the lowest energies.

**
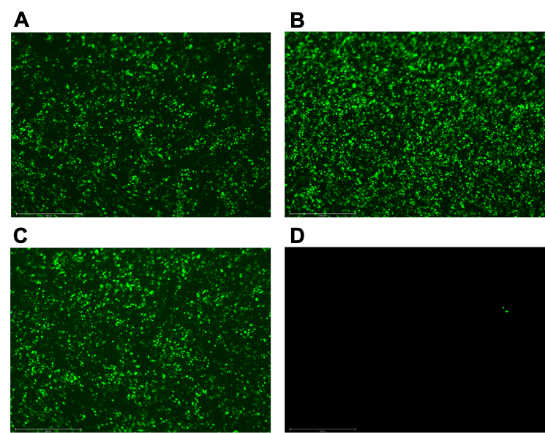
**

**Figure S11.** Fluorescent microscope images of GFP positive HEK 293T cells after 48hrs of SARS-CoV2 pseudovirus production (A). (B) Delta pseudovirus production. (C) Omicron pseudovirus production. (D) SARS-CoV2 pseudovirus infection against wild type HEK 293T. Scale bar equals 650 μm.


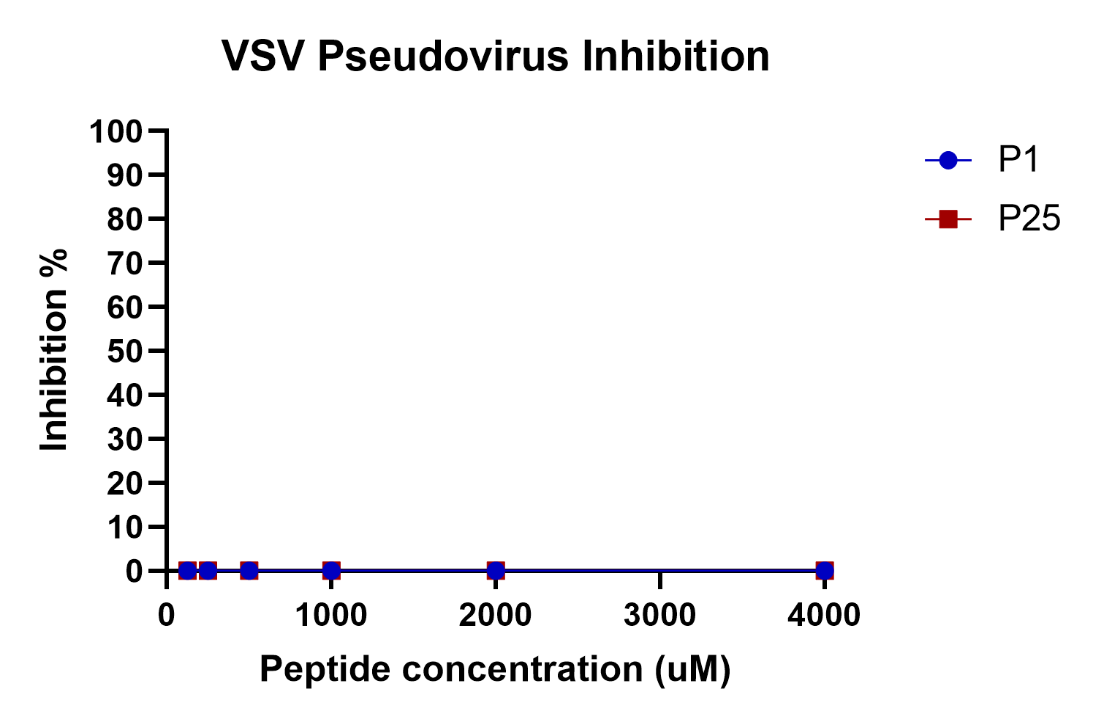


**Figure S12.** Neutralization assay of P1 and P25 peptides against Vesicular Stomatitis Virus (VSV) pseudovirus.

**Table S1.** List of primers for sequencing confirmation of Delta and Omicron pseudovirus variants.

| **SNo** | **Primer Name** | **Sequence** | **Length** | **Tm [^o^C]** | **GC%** |
| --- | --- | --- | --- | --- | --- |
| **1** | Delta 1 | gatgaggtgagacagattgcccctggacaa | 30 | 65 | 53 |
| **2** | Delta 2 | gacccacagaccttggagattctggacatc | 30 | 64 | 53 |
| **3** | Delta 3 | ggtgctcttctttggtccacacactgtggc | 30 | 68 | 57 |
| **4** | Delta 4 | cagaatctctgtggtcacagagatggtgaagtt | 33 | 64 | 45 |
| **5** | Omicron 1 | aggacccagctgcctcctgcttacaccaat | 30 | 69 | 57 |
| **6** | Omicron 2 | atctaccagacctctaatttcagagtgcag | 30 | 61 | 43 |
| **7** | Omicron 3 | acagagagcaacaagaagttcctgcctttc | 30 | 64 | 47 |
| **8** | Omicron 4 | attgccatccccacaaacttcaccatcagc | 30 | 66 | 50 |
| **9** | Omicron 5 | atcgctaatcagttcaacagcgccatcggaaag | 33 | 67 | 48 |
| **10** | Omicron 6 | ctgcactctgaaattagaggtctggtagat | 30 | 61 | 43 |
| **11** | Omicron 7 | gaaaggcaggaacttcttgttgctctctgt | 30 | 64 | 47 |
| **12** | Omicron 8 | gctgatggtgaagtttgtggggatggcaat | 30 | 66 | 50 |
| **13** | Omicron 9 | ctttccgatggcgctgttgaactgattagcgat | 33 | 68 | 48 |
| **14** | Omicron 10 | ctgatcagcgggtttaaacgggccctctag | 30 | 67 | 57 |

**Table S2.** Comparative summary of the main interactions from the spike of the original SARS-CoV2 and variants of concern against the designed peptides. Additionally, the other evaluated variants include Alpha, Beta, Gamma, Epsilon, and the presence of the mutation D614G.

| **Peptide** | **Spike origin** | **RBD conformational state** | **Interactions within RBD** | **Interactions outside the RBD** | **Interactions from peptide** |
| --- | --- | --- | --- | --- | --- |
| **P1** | SARS-CoV-2 | 1 RBD-up | ARG 403, LYS 417, TYR 453, GLN 493, GLN 498, THR 500, ASN 501, TYR 505 | - | GLN 1, LYS 3, THR 4, ASP 7, ASN 10, GLU 14, TYR 18 |
|  | Alpha | 1 RBD-up | GLY 545 | TYR 170, ASP 198, LYS 202, LEU 226, ASP 979, ARG 983 | LYS 3, THR 4, LYS 8, ASN 10, GLU 12, GLU 14, ASP 15, GLN 19 |
|  | Beta | 1 RBD-up | LYS 378, VAL 407, ASP 428 | - | GLN 1, ASP 7, LYS 8 |
|  | Delta | 1 RBD-up | - | LYS 41, ASP 198, TYR 200, ASP 228, GLN 755, ILE 973, ARG 983 | GLN 1, ALA 2, LYS 3, LYS 8, HIS 11, GLU 12, GLU 14 |
|  | D614G | 1 RBD-up | LYS 378, TYR 380, PRO 412, ASP 427, ASP 428 | - | LYS 3, ASN 10, GLU 14, TYR 18 |
|  | Gamma | 1 RBD-up | CYS 379, ARG 408, GLN 414 | - | ASP 15, LEU 22 |
|  | Epsilon | All RBD-down | LYS 378, GLN 414, ARG 408 | GLU 988, ASP 994, ARG 995 | GLN 1, ALA 2, THR 4, LYS 8, GLU 14, ASP 15, TYR 18 |
|  | Omicron | 1 RBD-up | GLY 416, TYR 421, ARG 457, TYR 473 | ASP 198, TYR 200, ASP 228 | GLN 1, LYS 3, THR 4, ASP 7, GLN 19, SER 21, LEU 22 |
| **P25** | SARS-CoV-2 | 1 RBD-up | ARG 403, LYS 417, ASP 420, TYR 449, GLN 493, ASN 460, TYR 505 | - | ILE 1, TRP 6, ASP 10, ASP 13, GLU 17, TYR 21 |
|  | Alpha | 1 RBD-up | PHE 377, LYS 378, SER 383, ARG 408 | - | ASP 10, GLU 15, ASP 18, GLN 22, SER 24 |
|  | Beta | 1 RBD-up | ALA 372, SER 375, PHE 377, LYS 378, ARG 408, ASN 437, TYR 508 | - | PHE 7, HIS 8, TYR 9, GLU 15, TRP 16, GLU 19, GLN 22 |
|  | Delta | 1 RBD-up | ARG 403, LYS 417, TYR 453, ASN 487, TYR 489, GLN 493 | - | ASP 10, HIS 14, GLU 19, GLN 22, SER 23 |
|  | D614G | 1 RBD-up | LYS 378, CYS 379, TYR 380, GLY 381, GLY 413, GLN 414, ASP 427 | - | HIS 8, LYS 11, TRP 12, GLU 15, TRP 16, GLU 19, SER 23 |
|  | Gamma | 1 RBD-up | LYS 378, ARG 408, GLN 414, THR 415 | - | ASP 10, ASP 13, GLU 17, TYR 21 |
|  | Epsilon | All RBD-down | TYR 380, ASP 405, VAL 407, ARG 408, GLY 413, GLN 414, THR 415, VAL 503, GLY 504 | ARG 995 | ILE 1, GLN 4, ASP 10, LYS 11, ASP 13, GLU 17, ASP 18, GLU 19, TRP 20, GLN 22, SER 24 |
|  | Omicron | 1 RBD-up | ASN 417, TYR 453, ARG 493, SER 496, ARG 498 | - | TYR 9, ASP 13, GLU 17, TRP 20, SER 24 |
